# One Juliet and four Romeos: VeA and its methyltransferases

**DOI:** 10.3389/fmicb.2015.00001

**Published:** 2015-01-20

**Authors:** Özlem Sarikaya-Bayram, Jonathan M. Palmer, Nancy Keller, Gerhard H. Braus, Özgür Bayram

**Affiliations:** ^1^Department of Biology, Maynooth University, National University of Ireland, Maynooth, Ireland; ^2^Center for Forest Mycology Research, Northern Research Station, United States Forest Service, Madison, WI, USA; ^3^Department of Medical Microbiology and Immunology, University of Wisconsin at Madison, Madison, WI, USA; ^4^Department of Molecular Microbiology and Genetics, Georg-August Universität Göttingen, Göttingen, Germany

**Keywords:** secondary metabolism, methyltransferases, velvet family, LaeA, VelB–VeA–LaeA, VosA, LlmF, VapA–VipC–VapB

## Abstract

Fungal secondary metabolism has become an important research topic with great biomedical and biotechnological value. In the postgenomic era, understanding the diversity and the molecular control of secondary metabolites (SMs) are two challenging tasks addressed by the research community. Discovery of the LaeA methyltransferase 10 years ago opened up a new horizon on the control of SM research when it was found that expression of many SM gene clusters is controlled by LaeA. While the molecular function of LaeA remains an enigma, discovery of the velvet family proteins as interaction partners further extended the role of the LaeA beyond secondary metabolism. The heterotrimeric VelB–VeA–LaeA complex plays important roles in development, sporulation, secondary metabolism, and pathogenicity. Recently, three other methyltransferases have been found to associate with the velvet complex, the LaeA-like methyltransferase F and the methyltransferase heterodimers VipC–VapB. Interaction of VeA with at least four methyltransferase proteins indicates a molecular hub function for VeA that questions: Is there a VeA supercomplex or is VeA part of a highly dynamic cellular control network with many different partners?

## INTRODUCTION

Small bioactive chemicals, also named secondary metabolites (SMs), are produced by several groups of organisms, including fungi, bacteria, and plants (Bok and Keller, 2004; Yu and Keller, 2005; Brakhage, 2013). Fungi are incredibly diverse and recent efforts in sequencing fungal genomes have demonstrated that they are rich in genes involved in production of SM. Additionally, it has been conservatively estimated that there are at least five million fungal species, of which there are only about 100,000 described species (O’Brien et al., 2005; Blackwell, 2011), thus the majority of fungi and their bioactive SMs remain unstudied. Genes encoding the enzymes responsible for the biosynthesis of SMs are often clustered in fungal chromosomes reminiscent of bacterial operons and are therefore often referred to as gene clusters. SM gene clusters tend to be transcriptionally co-regulated by a variety of different genetic mechanisms that range from specific regulation by DNA binding transcription factors to global regulation via changes in chromatin structure (Palmer and Keller, 2010; Strauss and Reyes-Dominguez, 2011; Gacek and Strauss, 2012). Interestingly, global regulatory protein complexes involved in fungal differentiation processes in response to environmental signals including light, nutrient deprivation or pH have also been shown to regulate SM gene clusters; solidifying the link between SM and fungal development.

Undoubtedly one of the few known conserved global regulators of SM in fungi is the putative methyltransferase LaeA (Bok and Keller, 2004). LaeA has been shown to control a large percentage of SM clusters as well as several aspects of fungal development in several genera (Kamerewerd et al., 2011; Butchko et al., 2012; Wu et al., 2012; Karimi-Aghcheh et al., 2013). Furthermore, LaeA forms a heterotrimeric protein complex with the two members of the fungal-specific velvet domain transcription factor family [VeA & a velvet-like protein B (VelB)] that coordinate fungal development and SM production (Bayram et al., 2008). Interestingly, in addition to LaeA, three more methyltransferases have recently been discovered to interact with VeA (Palmer et al., 2013; Sarikaya-Bayram et al., 2014). Given the fact that these complexes control many processes in fungal biology and it is the 10th anniversary of the discovery of LaeA, here we summarize recent insights into the role of the velvet complex and associated methyltransferases.

## VeA AS A MOLECULAR HUB FOR PROTEIN–PROTEIN INTERACTIONS

**FIGURE 1:**
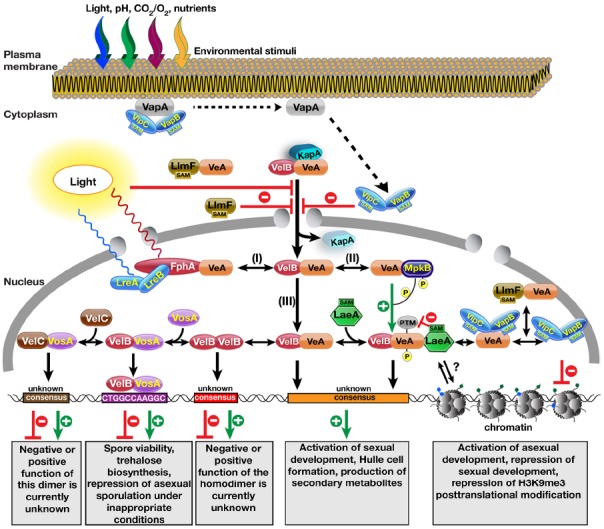
**Molecular complexes formed by the velvet family proteins and the methyltransferases on the control of fungal development and secondary metabolite production.** Nuclear entry of the VeA–VelB heterodimer is operated by the α-importin KapA protein. Light reduces nuclear entry of the heterodimer by an unknown mechanism. VapA tethers the two SAM-dependent methyltransferases to the plasma membrane. Reception of an unknown signal (e.g., light, pH, CO_2_, O_2_, starvation) triggers the release of VipC–VapB methyltransferase heterodimers, which are targeted to the nucleus. During their translocation to the nucleus, they inhibit the nuclear import of the VeA protein. Another LaeA-like SA\ependent methyltransferase LlmF also hinders the nuclear entry of the VeA protein and forms a complex with VeA. After entering the nucleus, the VelB–VeA dimer follows different options: (I) VeA interacts with the red light receptor protein phytochrome FphA, which together with blue light receptors LreA–LreB forms a tetrameric VeA–FphA–LreB–LreA complex. (II) VeA interacts with MAPK MpkB that phosphorylates VeA, which makes VeA more interactive for VelB. (III) VeA–VelB dimer is a part of more dynamic system where addition of methyltransferase LaeA results in the trimeric VelB–VeA–LaeA velvet complex. VelB–VeA or VelB–VeA–LaeA might bind to a consensus sequence to activate sexual developmental genes as well as SM gene clusters. Molecular function of LaeA between the chromatin and VelB–VeA heterodimer function is still unclear. VeA might recruit two methyltransferases VipC–VapB. Either VeA–VipC–VapB or VipC–VapB influence the histone posttranslational modifications (PTMs) and activates asexual genes. LlmF–VeA also forms a complex with VeA in the nucleus, whose function needs to be resolved. VelB component of VelB–VeA heterodimer dissociates from VeA by forming VelB–VelB homodimer. Free VelB also attracts the third velvet family protein VosA to form active transcription factor heterodimers that bind to target sequence of trehalose biosynthetic genes and asexual genes. Furthermore, VosA might recruit VelC, VosA–VelC heterodimer might activate the genes controlling sexual development and spore viability. Velvet family proteins might form more combinations of hetero and homodimer complexes as the Rel homology domain (RHD) proteins of NF-κB family in mammals.

The *veA* gene encodes a protein of 574 amino acid long, which is required for sexual development and SM production in *Aspergillus nidulans* (Kato et al., 2003; Bok et al., 2013). Deletion of *veA* leads to complete loss of sexual fruiting bodies, whereas an N-terminally truncated protein encoded by *veA1* results in a reduced cleistothecia and increased conidia production (Kim et al., 2002). With the availability of whole genome sequences and reverse-genetics, several other velvet-like proteins have been identified, which include VeA, VelB, VosA, and VelC (Bayram and Braus, 2012). The velvet family proteins are conserved in filamentous fungi, however they are absent in yeasts. VeA forms a heterotrimeric protein complex with VelB and the methyltransferase LaeA, which is required for the coordination of fungal development with SM production (Bayram et al., 2008). The trimeric velvet complex, VelB–VeA–LaeA, is predominantly formed in the nucleus when the fungus is grown without light, as VeA is translocated into the nucleus in the dark (Stinnett et al., 2007). Light is inhibitory to VeA expression as well as its nuclear entry through a currently unknown mechanism. VeA–VelB primarily enters into the nucleus together, where they meet the methyltransferase LaeA. Nuclear entry of the VeA–VelB heterodimer is driven by the α-importin KapA (Figure 1). VelB has additional functions in spore viability and trehalose biogenesis, which requires VelB–VosA heterodimer formation (Sarikaya Bayram et al., 2010).

While the velvet domain does not have amino acid similarity to other DNA binding domains, the different combinations of dimerization in the velvet proteins are reminiscent of the bZip family of transcription factors and as such were hypothesized to be transcription factors themselves. Biochemical characterization with the partial crystal structure of VosA–VelB heterodimer suggested that the velvet domain possesses a DNA binding fold similar to NF-κB transcription factors of mammals (Ahmed et al., 2013), and thus is potentially a transcription factor. NF-κB proteins in mammals are responsible for many cellular responses including antiviral, antibacterial responses of inflammation. VosA was shown to bind to an 11-nucleotide consensus sequence (CTGGCCAAGGC) found in the promoters of asexual regulators such as *brlA*, *wetA*, *vosA* as well as of trehalose biosynthetic genes *tpsA* and *treA*. Similarly, it was also found in the human pathogenic ascomycete *Histoplasma capsulatum* that VosA and VelB homologs (Ryp2 and Ryp3), are required for the dimorphic switch from hyphal form to a more pathogenic yeast form (Webster and Sil, 2008). Both Ryp2 and Ryp3 bind to a 10 nucleotide consensus sequence (A/T)CCA(T/C)GG(T/A)(T/A)(C/A) that is present in the promoters of the genes responsible for temperature-dependent dimorphic switch (Beyhan et al., 2013). VeA–VelB heterodimer presumably binds to DNA as well, however, they probably recognize a different consensus element than VelB–VosA because there are significant differences in the interaction surfaces of the velvet domains of VelB–VosA versus VeA–VelB. In analogy to Rel family proteins of NF-κB group, possible hetero- and homodimers among the velvet family proteins are likely to form within the nucleus, where they control different responses by binding to different consensus sequences. There is little known how the fourth velvet family protein VelC contributes to the development. VelC was originally described as a positive regulator of sexual development as VeA and VelB because deletion of *velC* gene in *A. nidulans* causes decreased number of cleistothecia and increased conidiation and might serve as an auxiliary factor of development in *A. nidulans* (Park et al., 2014). Interestingly, VelC was found to interact with VosA in yeast two-hybrid and *in vitro* studies, but, this interaction has not yet been confirmed *in vivo*. Although it is not known where VelC localizes, the VosA–VelC dimer might control development by binding to alternative promoter sequences (Figure 1). VelB forms homodimers *in vivo* and *in vitro* (Sarikaya Bayram et al., 2010). However, the function of these homodimers and their target consensus is currently unknown.

One of the proteins that VeA interacts with is a mitogen activated protein kinase (MAPK) AnFus3 (MpkB) that is a homologue of yeast Fus3 involved in the pheromone response pathway (Bayram et al., 2012). AnFus3 interacts with the VeA protein in the nucleus and phosphorylates VeA *in vitro*, which promotes formation of VeA–VelB heterodimer. In contrast, the VeA–LaeA interaction is not influenced by VeA phosphorylation (Bayram et al., 2012). Red light receptor phytochrome protein FphA also interacts with VeA protein, which together with the blue light receptors LreA–LreB forms the light complex (Purschwitz et al., 2008). In particular, VeA is in physical contact with the histidine kinase domain of the FphA, but VeA is not phosphorylated by the FphA protein (Purschwitz et al., 2009). It is currently unknown how the VeA–FphA–LreA–LreB complex acts at the molecular level to control light-dependent development and SM production.

These examples underscore the hypothesis that VeA acts as a molecular scaffold, thereby integrating several signals into the coordination of development and SM production ranging from MAPK signaling pathways to light receptors. However, an additional interesting facet of the VeA interacting proteins is that several of them are S-adenosyl L-methionine (SAM)-dependent methyltransferases, such as LaeA, LaeA-like methyltransferase F (LlmF), velvet interacting protein C (VipC), and VipC associated protein B (VapB). Given the central importance of the LaeA methyltransferase for secondary metabolism and development regulation, the link to these methyltransferases cannot be ignored, and thus these four interaction partners of the VeA will be further discussed.

## LaeA, A CRYPTIC AND ENIGMATIC METHYLTRANSFERASE

LaeA was the first methyltransferase discovered to associate with VeA. The *laeA* (*l*oss of *aflR e*xpression *A*) gene was originally identified through a forward genetics screen in *A. nidulans* looking for mutants that were unable to make precursors of the sterigmatocysin (ST; Butchko et al., 1999; Bok and Keller, 2004). Several of the isolated mutants had also lost expression of *aflR*, the ST cluster specific transcription factor. One of these mutants was then complemented with a genomic DNA cosmid library, which led to identification of the putative SAM-dependent methyltransferase LaeA (Bok and Keller, 2004). Subsequent generation of *laeA* null mutants (*ΔlaeA*) in *A. nidulans*, *A. fumigatus*, and *A. flavus*, demonstrated that LaeA was responsible for regulation of approximately 50% of the SM clusters in these species (Bok and Keller, 2004; Bok et al., 2005, 2006; Perrin et al., 2007; Kale et al., 2008; Georgianna et al., 2010). As noted above, this regulation of large number of SMs is conserved in other genera. The conserved regulation of SM clusters is also associated with reduced hyphal pigmentation as well as reduction in sporulation capacity. Besides SM production, LaeA also influences development by controlling the expression and posttranslational modifications (PTMs) of the velvet family proteins VeA, VelB, and VosA. In the absence of LaeA, protein levels of the velvet family elevate and VeA undergoes an unknown PTM, which results in drastically reduced Hülle cell formation and small cleistothecia (Sarikaya Bayram et al., 2010; Shaaban et al., 2010).

To date, all examined *ΔlaeA* mutants of pathogenic fungi have been found to display reduced virulence. This was first described in the human pathogen *A. fumigatus* (Bok et al., 2005) and later for plant pathogenic fungi including *A. flavus* (Kale et al., 2008; Amaike and Keller, 2009), *Cochliobolus heterostrophus* (Wu et al., 2012), and several *Fusarium* species (Wiemann et al., 2010; Butchko et al., 2012; Lopez-Berges et al., 2013). Several of the SMs regulated by LaeA in these species contribute to virulence or cellular damage as described most thoroughly in studies of *A. fumigatus* where helvolic acid, gliotoxin, endocrocin, pseurotin, hexadehydro-astechrome, fumagillin, and fumitremorgin have all been found to be regulated by LaeA (reviewed in, Jain and Keller, 2013; Wiemann et al., 2013).

Thus far, all the research done on LaeA in filamentous fungi is suggestive of an integral role in mediating fungal development and SM production, however the obvious question remains, “what is the molecular function of LaeA?” Due to LaeA harboring a required methyltransferase domain and coupled with gene expression studies illustrating the regulation of the contiguous gene clusters, several groups have hypothesized that LaeA could be regulating SM clusters epigenetically through modification of chromatin structure, either directly or indirectly. The first evidence for this comes from a microarray study in *A. fumigatus* (Perrin et al., 2007), where Perrin et al. (2007) showed that LaeA influences the expression of 9.5% of the *A. fumigatus* genome, where 13 out of 22 SM gene clusters were strongly down regulated in the absence of *laeA*. Further clues regarding the LaeA involvement in epigenetic control come from a study where it was shown that lack of LaeA leads to more heterochromatin (HepA) occupancy with increased repressive histone 3 (H3) lysine 9 (K9) trimethylation (H3K9me3) in the ST gene cluster (Reyes-Dominguez et al., 2010). In addition to increased HepA and H3K9me3 accumulation, the levels of canonical activation marks decreased (H3K9/14 acetylation). Similar to the study in *A. nidulans*, but at a less pronounced level, 75 genes of *Trichoderma* showed a LAE1-regulated pattern for their expression correlating with changes in histone marks (H3K4me3; Karimi-Aghcheh et al., 2013). While these data suggest that chromatin structure is changed in *ΔlaeA* mutants, they do not directly link LaeA to modification of histones and subsequent epigenetic control. Significant efforts have been made to find a methylation substrate of LaeA, however a recent biochemical study was not able to identify a substrate *in vivo* nor *in vitro* (Patananan et al., 2013). Interestingly, a novel automethylation reaction producing S-methylmethionine was discovered in LaeA, however subsequent mutagenesis of this residue resulted in a functional LaeA protein (Patananan et al., 2013). Thus, if LaeA has another methylation substrate besides itself, whether it might be a demethylase and requires methylated substrates or how LaeA controls SM cluster regulation remains an enigma.

## LaeA-LIKE METHYLTRANSFERASE LlmF

While the molecular function of LaeA remains unknown, there are several other putative methyltransferases that have homology to LaeA in fungal genomes. These proteins have recently been studied in *A. nidulans* and named LaeA-like methyltransferases (LlmA–LlmJ; Palmer et al., 2013). These nine genes were systematically deleted to determine their roles in development and SM production. Interestingly, LlmF was found to control production of ST as well as influence sexual development. Moreover, LlmF was shown to interact with VeA in a yeast-two-hybrid assay and that interaction was confirmed *in vivo*. While LaeA is constitutively localized in the nucleus, LlmF exhibited a nucleo-cytoplasmic distribution. LlmF is hypothesized to function by directly interacting with VeA and influencing the nuclear/cytoplasmic ratio of VeA. In the absence of LlmF, VeA accumulates in the nucleus, which results in increased production of ST and increased sexual development. Consistently, over-production of LlmF results in VeA accumulation in the cytoplasm and thus decreased sexual development and ST production (Palmer et al., 2013). Biochemical analysis showed that the SAM-binding motif is required for LlmF function, however none of the proteins known to be involved in nuclear transport of VeA (VeA, VelB, or KapA) could be identified as a methylation substrate. Although it was proposed that LlmF–VeA interaction takes place in the cytoplasm, it is still possible that LlmF might also interact with VeA within the nucleus since LlmF is not completely excluded from the nuclear fraction (Figure 1).

A homologue of LlmF was also studied in the maize pathogen *C. heterostrophus* that produces the host-selective T-toxin. In contrast to the *laeA1* and *vel1* mutants where T-toxin production decreases, deletion of *llm1* gene in this fungus leads to increased T-toxin production, suggesting a repressive role for Llm1 in the production of this toxin in *C. heterostrophus* (Bi et al., 2013). This repressor role of Llm1 on T-toxin is similar to the role of LlmF on ST production in *A. nidulans*. In agreement with deletion studies, overexpression of *llm1* represses T-toxin production and similar to *A. nidulans*, *llm1* is not epistatic to *laeA1* and *vel1*. Furthermore, overexpression of *llm1* results in individuals that cannot act as fertile females and de-repression of asexual sporulation during sexual as well as vegetative growth.

## THE HETEROTRIMERIC ZINC FINGER-METHYLTRANSFERASE COMPLEX VapA–VipC–VapB

An experimental strategy followed by Sarikaya-Bayram et al. (2014) led to the discovery of the third VeA interacting methyltransferase, VipC, also named LlmB. Detailed biochemical analysis of the VipC interaction partners resulted in discovery of two VipC associated proteins (VapA, VapB) with VapB representing the fourth VeA interacting methyltransferase. The heterotrimeric VapA–VipC–VapB complex controls the appropriate morphogenic responses to environmental stimuli such as light.

VapA is a FYVE zinc finger protein that acts as a negative regulator of asexual conidiation and as positive regulator of sexual development. Deletion of *vapA* leads to increased conidiation and reduced cleistothecia production, and consistently the transcripts of asexual regulators, BrlA and AbaA, are substantially increased in the *vapA* deletion strain. The VapA protein is localized to the plasma membrane as the other FYVE type zinc finger proteins (Hayakawa et al., 2007). The major function of VapA protein is to recruit the two methyltransferase components of the complex, VipC and VapB, to the plasma membrane and not to release them until an environmental signal triggers their release.

VipC–VapB heterodimer is a negative regulator of sexual and positive regulator of asexual development. This function of VipC–VapB is similar to the function of LlmF on development. Both VipC and VapB proteins are found at the plasma membrane as well as in the nuclear fraction. bimolecular fluorescence complementation (BIFC) showed that VipC–VapB interacts with the zinc finger VapA at the plasma membrane, but VipC–VapB heterodimer is also found in the nucleus.

Interestingly, similar to LaeA and LlmF, this small methyltransferase heterodimer also interacts with VeA protein and this interaction takes place around and inside the nucleus. Moreover, they influence the developmental responses of the fungus in part by modulating nuclear accumulation of the VeA protein, which is similar to the LlmF effect whereas the VipC–VapB heterodimer negatively influences VeA nuclear entry, the antagonistically acting VapA positively affects VeA nuclear accumulation. This positive impact on VeA nuclear localization is not a direct result of VapA because zinc finger VapA and transcription factor VeA do not interact *in vivo*. VapA supports VeA localization by keeping VipC–VapB heterodimers at the plasma membrane and preventing their membrane-cytoplasm-nucleus migration. Recently, it has been shown by Bok et al. (2013) that VeA acts as a repressor of the polyketide orsellinic acid (OA) gene cluster. In the absence of *veA*, the metabolites OA and its derivatives, F9775A-B are produced in higher amounts and mycotoxin ST is not produced. Confirming these results, overexpression of *vapB* methyltransferase leads, similarly, to high OA levels and lack of ST, which is caused by the negative effect of VapB methyltransferase on VeA nuclear accumulation as well as transcription.

How does the VapA–VipC–VapB complex control asexual gene expression? VapA protein does not show DNA binding zinc finger features. Furthermore, it is not found to influence transcriptional machinery in the nucleus. However, the VipC–VapB heterodimer is found in the nucleus and has the potential to control gene expression. The N-terminus of histone proteins is highly conserved in eukaryotes and undergoes various PTMs such as acetylation, methylation, phosphorylation, sumoylation, and ubiquitinylation. Histone 3 is the most intensively modified histone protein among the other histones. Especially, acetylation of histone 3 lysine 9 (H3K9ac) leads to gene activation whereas methylation of the same residue H3K9me2 (dimethylated) or me3 (trimethylated) results in gene silencing by attracting the heterochromatin protein responsible for heterochromatin formation. First hints for the nuclear function of the VipC–VapB methyltransferases come from their influence on histone PTM. Particularly, VapB overexpression leads to almost 50% reduction in H3K9me3 levels, suggesting that VapB is involved in control of gene expression by epigenetic interference. In connection with the reduced H3K9me3 marks, nuclear distribution of the heterochromatin protein (HepA) drastically alters. Furthermore, repressive H3K9me3 increases in the *abaA* promoter when VipC–VapB are absent but diminishes in *vapA* mutant strain. These data draw a picture where VipC and particularly VapB counteracts against repressive H3K9me3 with an unknown mechanism. Similarly to the effect of LaeA on chromatin, it is currently unknown whether this is a direct influence of VapB, e.g., as demethylase or the result of indirect interactions of VapB with several regulators such as the VeA protein.

VapA–VipC–VapB orthologs are highly conserved in the fungal kingdom as are the LaeA and LlmF methyltransferases, which suggests that this heterotrimeric complex might possess major regulatory roles in fungal development, virulence, and SM production in the other members of the fungal kingdom. There are already hints for the similar complexes in fungi such as *Fusarium graminearum* where several Vips were found as FgVeA interaction partner in a yeast two-hybrid screen in this fungus (Jiang et al., 2011). One out of the six identified Vip proteins is a LaeA homolog protein, the other five Vips are all methyltransferase domain containing proteins similar to LlmF and VipC (LlmB). However, deletions of these genes do not lead to serious consequences on virulence or the SM production of *F. graminearum*.

Interaction of the VeA protein with at least four methyltransferases (LaeA, LlmF, VipC, VapB) suggests that VeA should have an affinity domain for these methyltransferases. However, such an affinity domain has not been shown except for the LaeA–VeA interaction where LaeA binds to C-terminus of VeA (Bayram et al., 2008). Furthermore, detailed multiple alignments of the four methyltransferases exhibit only conserved regions around the SAM binding domain whereas the N as well as C terminal domains differ from each other. These data indicate that perhaps a tertiary domain found in VeA provides the interaction between the VeA protein and the methyltransferases. Future structural studies will show whether this assumption is correct or whether there are completely different mechanisms how VeA attracts methyltransferases.

## CONCLUSION

The fungal kingdom has an estimated five million species and thus represents an enormous amount of potential in terms of discovering pharmaceutically important small molecules. With the advances in whole genome sequencing, the number of SM identified from fungi has been rapidly increasing. The regulatory complexes governing the SM clusters are key to our ability to understand the biological roles of SM. Whereas many regulatory players of development and secondary metabolism have been identified, the exact molecular mechanisms that drive the fungal growth and SM production are still in their infancy. While the molecular function of LaeA remains an enigma, research on this topic has greatly contributed to our understanding of gene cluster regulation as well as the link between development and secondary metabolism. The founding member of the velvet family, VeA, appears to be immersed in the core of the fungal transcriptional response to environmental stimuli, which is evidenced by its plethora of interacting proteins. Novel players such as LlmF, VipC, and VapB, which were discovered to be in connection with the velvet complex, have been increasing the complexity of the general picture and suggesting the presence of a VeA supercomplex. Understanding the mechanistics of these complicated but highly orchestrated transcriptional response is crucial to strengthen our knowledge on SM production and fungal development, which will allow us to use the power of fungi in medicine, agriculture, and biotechnology.

### Conflict of Interest Statement

The authors declare that the research was conducted in the absence of any commercial or financial relationships that could be construed as a potential conflict of interest.
